# Informing Developmental Milestone Achievement for Children With Autism: Machine Learning Approach

**DOI:** 10.2196/29242

**Published:** 2021-06-08

**Authors:** Munirul M Haque, Masud Rabbani, Dipranjan Das Dipal, Md Ishrak Islam Zarif, Anik Iqbal, Amy Schwichtenberg, Naveen Bansal, Tanjir Rashid Soron, Syed Ishtiaque Ahmed, Sheikh Iqbal Ahamed

**Affiliations:** 1 R.B. Annis School of Engineering University of Indianapolis Indianapolis, IN United States; 2 Ubicomp Lab, Department of Computer Science Marquette University Milwaukee, WI United States; 3 College of Health and Human Sciences Purdue University West Lafayette, IN United States; 4 Department of Mathematical and Statistical Sciences Marquette University Milwaukee, WI United States; 5 Telepsychiatry Research and Innovation Network Ltd Dhaka Bangladesh; 6 Department of Computer Science University of Toronto Toronto, ON Canada

**Keywords:** autism spectrum disorders, machine learning, digital health, mobile health, mhealth, predictive modeling, milestone parameters, Autism and Developmental Disabilities Monitoring (ADDM), early intervention

## Abstract

**Background:**

Care for children with autism spectrum disorder (ASD) can be challenging for families and medical care systems. This is especially true in low- and- middle-income countries such as Bangladesh. To improve family–practitioner communication and developmental monitoring of children with ASD, mCARE (Mobile-Based Care for Children with Autism Spectrum Disorder Using Remote Experience Sampling Method) was developed. Within this study, mCARE was used to track child milestone achievement and family sociodemographic assets to inform mCARE feasibility/scalability and family asset–informed practitioner recommendations.

**Objective:**

The objectives of this paper are threefold. First, it documents how mCARE can be used to monitor child milestone achievement. Second, it demonstrates how advanced machine learning models can inform our understanding of milestone achievement in children with ASD. Third, it describes family/child sociodemographic factors that are associated with earlier milestone achievement in children with ASD (across 5 machine learning models).

**Methods:**

Using mCARE-collected data, this study assessed milestone achievement in 300 children with ASD from Bangladesh. In this study, we used 4 supervised machine learning algorithms (decision tree, logistic regression, K-nearest neighbor [KNN], and artificial neural network [ANN]) and 1 unsupervised machine learning algorithm (K-means clustering) to build models of milestone achievement based on family/child sociodemographic details. For analyses, the sample was randomly divided in half to train the machine learning models and then their accuracy was estimated based on the other half of the sample. Each model was specified for the following milestones: *Brushes teeth, Asks to use the toilet, Urinates in the toilet or potty, and Buttons large buttons*.

**Results:**

This study aimed to find a suitable machine learning algorithm for milestone prediction/achievement for children with ASD using family/child sociodemographic characteristics. For *Brushes teeth*, the 3 supervised machine learning models met or exceeded an accuracy of 95% with logistic regression, KNN, and ANN as the most robust sociodemographic predictors. For *Asks to use toilet,* 84.00% accuracy was achieved with the KNN and ANN models. For these models, the family sociodemographic predictors of “family expenditure” and “parents’ age” accounted for most of the model variability. The last 2 parameters, *Urinates in toilet or potty* and *Buttons large buttons*, had an accuracy of 91.00% and 76.00%, respectively, in ANN. Overall, the ANN had a higher accuracy (above ~80% on average) among the other algorithms for all the parameters. Across the models and milestones, “family expenditure,” “family size/type,” “living places,” and “parent’s age and occupation” were the most influential family/child sociodemographic factors.

**Conclusions:**

mCARE was successfully deployed in a low- and middle-income country (ie, Bangladesh), providing parents and care practitioners a mechanism to share detailed information on child milestones achievement. Using advanced modeling techniques this study demonstrates how family/child sociodemographic elements can inform child milestone achievement. Specifically, families with fewer sociodemographic resources reported later milestone attainment. Developmental science theories highlight how family/systems can directly influence child development and this study provides a clear link between family resources and child developmental progress. Clinical implications for this work could include supporting the larger family system to improve child milestone achievement.

## Introduction

### Background

Autism spectrum disorder (ASD) is a global problem [1] and a heterogeneous neurodevelopmental disorder [2]. In 1943, Kanner [3] first described this disorder in children’s behavior [3]. In this neurodevelopmental disorder, children have social communication issues, repetitive behaviors, restrictive interests, and professional impairments throughout their lifespan [4,5]. In developed countries, 1%-1.5% of children have ASD [4], whereas in the United States, 1 out of 54 children have ASD [6,7]. Although it is the fastest growing developmental disorder, the number of individuals affected globally remains largely unknown [8]. In low- and middle-income countries, this rate is estimated to vary between 0.15% and 0.8%, whereas in a developing country such as Bangladesh this rate is reported to be 3% [9-11]. ASD symptoms gradually show up before 1 year of age, with nearly 80% of problems being identified by 2 years of age [12,13]. In particular, boys are affected 3 to 4 times more than girls with ASD [14]. Unfortunately, nearly 46% of children with ASD do not receive the proper treatment following diagnosis [8].

Medically, early identification and diagnosis of ASD will improve positive functional outcomes in later life for these children [15-18]. As a result, in 2000, the American Academy of Neurology and Child Neurology recommended to screen every child for ASD [14,19-21]. In other words, a reliable ASD diagnosis should be performed in children before 24 months of age [19], as this substantially improves the opportunities for recovery and also reduces the burden on caregivers (diagnostic odyssey) [16]. The major barriers to making improvements in ASD diagnosis and treatment are lack of proper knowledge about ASD, lack of motivation and patience of parents or caregivers, and delayed identification and diagnosis of ASD. Early identification and diagnosis help the care practitioners to make evidence-based decisions during intervention, which has both positive and long-term outcomes on the improvement of patients with ASD [5,22]. Physical therapy or exercise is much more important than medicine in the development of many patients with ASD, and in such cases early intervention can play an important role [23-26].

Besides the early identification and diagnosis, parents’ or caregivers’ demography, social or environmental demography, race, and ethnicity can play a vital role in the developmental process of children with ASD [15,27-31]. Concerning parents’ demography, educational level, occupation, family income and expenditures, number of siblings, and living area remain very important factors in the development of children with ASD [27-30]. Environmental factors such as the socioeconomic condition, neighborhood, and society’s attitudes toward children with ASD are very significant [12,13]. Although genes increasing the risk for ASD in children are mostly prenatal [32], demography of parents remains very important [33], as it can affect the improvement of patients with ASD. In this study, we will use the parents’, environmental, and social demography as a parameter to develop a machine learning model for predicting the improvement level of milestone parameters in children with ASD.

Based on the demography, machine learning models can predict the milestone parameters in children with ASD during their early intervention period. In this study, we have used 10 important demographic information in 4 supervised machine learning models to predict the improvement level of “daily living skills.” In the “Decision Tree” [34] machine learning algorithm, we have used the “Classification Trees” category to build the predictive model. To build a statistical model for our binary dependent variable, we deployed “Logistic Regression” with the sigmoid function [35,36] as the logistic function. We then deployed our preprocessed data sets in the K-nearest neighbor (KNN) algorithm using the “Euclidean distance” [37] to find the nearest neighbor. In the end, we used an artificial neural network (ANN) to build our last predictive model. In ANN, we have used “relu” as the hidden layer’s activation function, and “sigmoid” as the output layer’s activation function.

### Prior Work

In our previous work (Mobile-Based Care for Children with Autism Spectrum Disorder Using Remote Experience Sampling Method [mCARE]), we developed a mobile-based system to regularly monitor children with ASD with the help of caregivers in Bangladesh. In mCARE, we deployed a remote experience sampling method to monitor the milestone and behavioral parameters. These longitudinal data can be used in the intervention process, where the care practitioners can make evidence-based decisions based on the data. This tool was very effective in the development process of children with ASD; using this tool, the caregiver and care practitioner can observe the improvement level over a certain period on a graphical view. This tool not only assists the care practitioners but also motivates the caregivers. Besides, this tool has some renowned applications and studies to assist with the ASD diagnosis process in different phases [2,15,19,38,39]. While most studies have been performed for the early identification or recognition of ASD [16,19,22,40-44], little work has been done so far on the prediction of improvement level of ASD parameters or the timeframe for a certain level of improvement, or on the factors that need to be improved. In this study, we developed a relationship between the parents’ demography and the improvement in ASD milestone parameters by deploying a real data set of the mCARE system.

### Goal of This Study

Demographic data such as family income, living place, facilities, parents’ age, education and occupation, family types, and number of siblings affect parental stress and psychology [45]. This parental stress and psychological stress definitely impact the mental development of children with ASD, especially “daily living skills” [46-48]. For this reason, cognitive behavioral therapy is very effective on the daily living skill development of children with ASD [49]. In this study, our main goal was to predict the improvement in an ASD milestone parameter (ie, daily living skills) using a machine learning algorithm based on demographic data of caregivers. To achieve our goal, first, we measure the improvement level of the milestone in children with ASD from the mCARE tools. Second, we will deploy an mCARE data set in 5 supervised and 1 unsupervised machine learning algorithm to build the best milestone parameters improvement prediction model. Finally, we will describe the importance of the caregiver-specific demography in predicting the improvement level of certain milestone parameters in children with ASD.

## Methods

### mCARE

**Figure 1:**
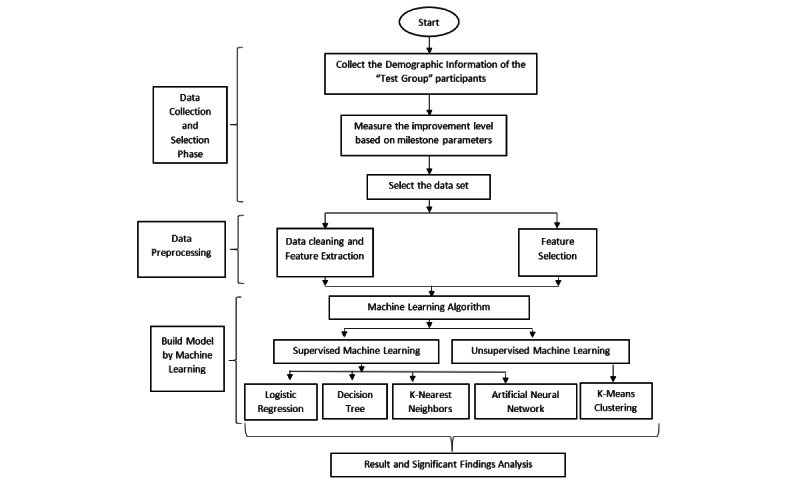
Outline of research design.

mCARE is a mobile-based app for monitoring the milestone and behavioral parameters of children with ASD regularly and remotely. This project was awarded by the National Institutes of Health (NIH) [50] and has been implemented in Bangladesh for 2 years. For this study, we used data from the mCARE study, which was approved by the Institutional Review Board of the Marquette University on July 9, 2020 (protocol number HR-1803022959). The mCARE study recruited 316 participants, of which we recruited 300 for this study. We deployed the remote experience sampling method to collect data on children with ASD, which was achieved by their caregiver using a smartphone app or an SMS text message. This mobile-based app has significance in the mental health intervention process, where by using the mCARE: Data Management Portal (mCARE: DMP), a caregiver can observe the longitudinal behavioral or milestone data graphically for a certain period. This feature helped the caregiver to make evidence-based decisions in the intervention process. In this study, we will first measure the improvement level of the “test group” participants based on milestone parameters. Using the test group data set, we will build the machine learning–based prediction model for a specific milestone parameter. We will use the test group patients’ demography for constructing the prediction model. Figure 1 summarizes the research design in a simple flowchart.

### Data Collection and Selection Phase

Following approval from the Marquette University Institutional Review Board (Protocol number HR-1803022959), the mCARE project recruited a total of 300 children with ASD (aged 2-9) from Bangladesh. We incorporated diversity in terms of age, sex, ASD severity, and family socioeconomic recourses. We divided the whole sample population into 2 groups: (1) the test group and (2) the control group. Patients in the test group were intervened and monitored regularly, whereas those in the control group were monitored over a certain period. Data from the control group and the test group were compared. This study took place in 4 major institutes of Bangladesh located in 2 geographical locations (Dhaka and Chittagong). We collaborated with 2 government organizations for ASD treatment and research, namely, The National Institute of Mental Health (NIMH) [51] and The Institute of Pediatric Neuro-disorder & Autism (IPNA) [52], to recruit 100 caregivers of children with ASD from each. The participants from each organization were divided into 2 groups: mCARE-APP (n=50) and mCARE-SMS (n=50). Each group was further divided equally into the test (n=25) and control (n=25) groups. Typically, in Bangladesh, families with low and high socioeconomic status receive treatment from public and private organizations, respectively. Therefore, to include participants from all socioeconomic classes, we included 2 private organizations, namely, Nishpap [53] and Autism Welfare Foundation (AWF) [54]. A total of 50 participants chosen from each of these schools were divided into the test group (n=25) and the control group (n=25) only for the mCARE-APP study group. The patient distribution among the 4 centers and the participant demography are presented in Tables 1 and 2, respectively.

**Table 1:** Patient distribution among the 4 centers.

Serial	Center name	Patients distribution	
		Test group (n=150)	Control group (n=150)
1	The National Institute of Mental Health (NIMH)	50	50
2	The Institute of Pediatric Neuro-disorder & Autism (IPNA)	50	50
3	Autism Welfare Foundation (AWF)	25	25
4	Nishpap Autism Foundation	25	25

**Table 2:** Demographic information of participants in the test group (n=150).

Demographics		mCARE: test group, n (%)
**Age (years)**		
	2-6	37 (24.7)
	6-9	113 (75.3)
**Sex**		
	Male	124 (82.7)
	Female	26 (17.3)
**Education of children**		
	Never went to school	34 (22.7)
	Went to usual academic school but failed to continue study	22 (14.7)
	Went to specialized school but failed to continue study	4 (2.7)
	Currently he/she is going to usual academic school	12 (8.0)
	Currently he/she is going to specialized academic school	78 (52.0)
**Father’s education**		
	Primary	29 (19.3)
	Secondary	23 (15.3)
	Undergraduate	23 (15.3)
	Graduate	29 (19.3)
	Postgraduate	46 (30.7)
**Mother’s education**		
	Primary	19 (12.7)
	Secondary	37 (24.7)
	Undergraduate	25 (16.7)
	Graduate	32 (21.3)
	Postgraduate	37 (24.7)
	Student	0.0 (0.0)
	Unemployed	4 (2.7)
**Father’s occupation**		
	Service	70 (46.7)
	Business	45 (30.0)
	Cultivation	1 (0.7)
	Other	7 (4.7)
	Unemployed	23 (15.3)
**Mother’s occupation**		
	Student	0.0 (0.0)
	Unemployed	0.0 (0.0)
	Housewife	124 (82.7)
	Service	17 (11.3)
	Business	4 (2.7)
	Cultivation	0 (0.0)
	Maid	1 (0.7)
	Other	1 (0.7)
	Not applied	3 (2.0)
**Average family spending per month (in thousand Taka)^a^**		
	<15 K	19 (12.7)
	15-30 K	44 (29.3)
	30-50 K	31 (20.7)
	>50 K	56 (37.3)
**Family type**		
	Nuclear	113 (75.3)
	Extended	37 (24.7)
**Geographic location**		
	Urban	120 (80.0)
	Semiurban	15 (10.0)
	Rural	15 (10.0)
	Slum	0.0 (0.0)

^a^US $1=84.77 Taka (as of March 18, 2021).

### Demographic Information of the Participants in the “Test Group”

We collected demographic information about participants in the test group (n=150). In Table 2, we present in detail the demographic information of participants that took part in the mCARE study.

### Measuring the Improvement Level Based on Milestone Parameters

In the mCARE project, there were 4 types of milestone for every test group patient. These were “daily living skills,” “communication,” “motor skills,” and “socialization.” Further, for every patient, based on his/her condition, the recruited care practitioner set different types of parameter from every milestone category. Table 3 lists the 4 types of parameters from each milestone group along with the participant numbers (n). Here the participant number (n) is different for different milestone parameters, as every participant did not have the same milestone parameter initially set by the care practitioner. At the beginning of this project, the care practitioners obtained the baseline information for every milestone parameter by screening the participant. Then, in the project timeline (2 years), the caregiver continuously updated the milestone parameter using the mCARE: APP or mCARE: SMS tool based on the child’s condition. At the end of the project, one can generate the participant’s end improvement level for different levels of their milestone parameters. By comparing the baseline milestone data with the end participant’s improvement data, we can calculate the improvement level (in percentage) for every milestone parameter (described in Table 3). In this table, besides the improvement level, we calculated the 95% CI for the validation of our results. As our sample size was 150, we used the Z value (1.96 for 95% CI) [55] for calculating the 95% CI using the following formula:


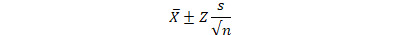


where 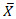 is the mean, *Z* is 1.96 (chosen from the Z-value table [55]), *S* is the SD, and *n* is the average sample number.

**Table 3:** Improvement level of the test group (mCARE) on their milestone parameters.

Milestone type and parameter with total participants (n)		Improvement level (%)	95% CI^a^				
			Average sample (n)		Lower-upper bound		Average improvement
**Daily living skills**			117		77.88-86.12		82
	Asks to use toilet (n=106)	61 (57.5)					
	Brushes teeth (n=140)	113 (80.7)					
	Buttons large buttons in front, in correct buttonholes (n=109)	70 (64.2)					
	Urinates in toilet or potty (n=113)	84 (74.3)					
**Communication**			90		33.48-42.01		37.75
	Listens to a story for at least 15 minutes (n=101)	35 (34.7)					
	Points to at least five body parts when asked (n=117)	62 (52.9)					
	Says month and day of birthday when asked (n=116)	42 (36.2)					
	Says own phone number when asked (n=23)	12 (52.1)					
**Motor skills**			123		80.94-86.06		83.5
	Draws circle freehand while looking at an example (n=136)	100 (73.5)					
	Glues or pastes 2 or more pieces together (n=130)	87 (66.9)					
	Jumps with both feet off the floor (n=104)	65 (62.5)					
	Runs smoothly without falling (n=119)	82 (68.9)					
**Socialization**			96		32.58-45.42		39
	Ends conversation appropriately (eg, “good bye” or “khoda hafez”) (n=14)	4 (28.5)					
	Keeps comfortable distance between self and others in social situations (n=130)	76 (58.4)					
	Talks with others about shared interests (eg, sports, TV shows, cartoons) (n=126)	50 (39.6)					
	Uses words to express emotions (eg, “I am happy” or “I am scared”) (n=110)	24 (21.8)					

### Data Set Selection

In the mCARE study, among the 4 categories (Table 3) of milestone parameters, the “daily living skills” showed the highest improvement level. In this study, we selected this category for building the prediction model based on the participant’s demography. In this milestone type, there are 4 different parameters: *Asks to use toilet*, *Brushes teeth*, *Buttons large buttons in front, in correct buttonholes*, and *Urinates in toilet or potty*. We took the demographic information for every participant who had these milestone parameters and created 4 data sets. In each data set, there were 18 features regarding the participant’s demographic (Multimedia Appendix 1) and 1 value for the “end improvement level” for each participant (this is the label value that will be used in supervised machine learning). We titled each data set by the name of the milestone parameter; for example, *Asks to use toilet*, which has 106 instances; *Brushes teeth*, which has 140 instances; *Buttons large buttons in front, in correct buttonholes*, which has 109 instances, and *Urinates in toilet or potty*, which has 113 instances. In the following sections, we describe the different machine learning models based on these 4 data sets.

### Data Preprocessing

Before building the prediction model, we have preprocessed our data set into 3 steps. In the following section, we will describe these steps.

### Data Cleaning and Feature Extraction

In the data cleaning step, we observed some missing data, especially with regard to age and salary, in our data sets. We handled this by replacing the empty cell with the mean value for that particular data set. In our data sets, out of 19 columns, only 6 had a numerical value, whereas others had a string input. Therefore, we created dummy variables for every column and converted the string input into a numerical input to handle this. For example, we categorized the column “gender” into 2 subcolumns, namely, “male” and “female.” The corresponding binary codes were set as “1” if the original input is male; otherwise “0.” By using a similar approach we set the female column. We could thus convert our whole data set into a numeric type by this feature extraction, but the problem is it increased the feature number to 48 from 19. Besides the feature extraction, we used the MinMaxScaler [56,57] to convert all of our features from the 0 to 1 range, as it increases the performance of the machine learning algorithm [58].

### Feature Selection

To get the most important features, we first created an extended data set from the “daily living skills” parameter with 18 features. We have used 3 different feature selection methods (univariate selection [59,60], feature importance [59,61-63], and correlation matrix with heatmap [59,64]) with our domain knowledge to select the 10 most important features from the extended data sets. From univariate selection [60] and feature importance [61-63], we obtained 10 important features with their score from each approach (Multimedia Appendices 2 and 3, respectively). We also prepared an important correlation matrix (Multimedia Appendix 4) with heatmap [64] for the features. After computing the most important features with their scores from the 3 feature selection methods, we selected the 10 most important features using these results and our domain knowledge. These features were “family expenditure,” “mother age,” “father age,” “going to specialized school,” “number of siblings,” “housewife-mother,” “father in service,” “living in urban,” “nuclear family,” and “mother education level (undergraduate).” After that, we again split the extended data set into 4 data sets (ie, Brushes teeth; Buttons large buttons in front, in correct buttonholes; Urinates in toilet or potty; and Asks to use toilet) using only these 10 features and with the “end improvement level.” These feature-selected data sets are very important in machine learning algorithm to boost up model performance.

### Exploring the Relationship and Associations Underlying the Data Set by Unsupervised Machine Learning: K-Means Clustering

**Figure 2:**
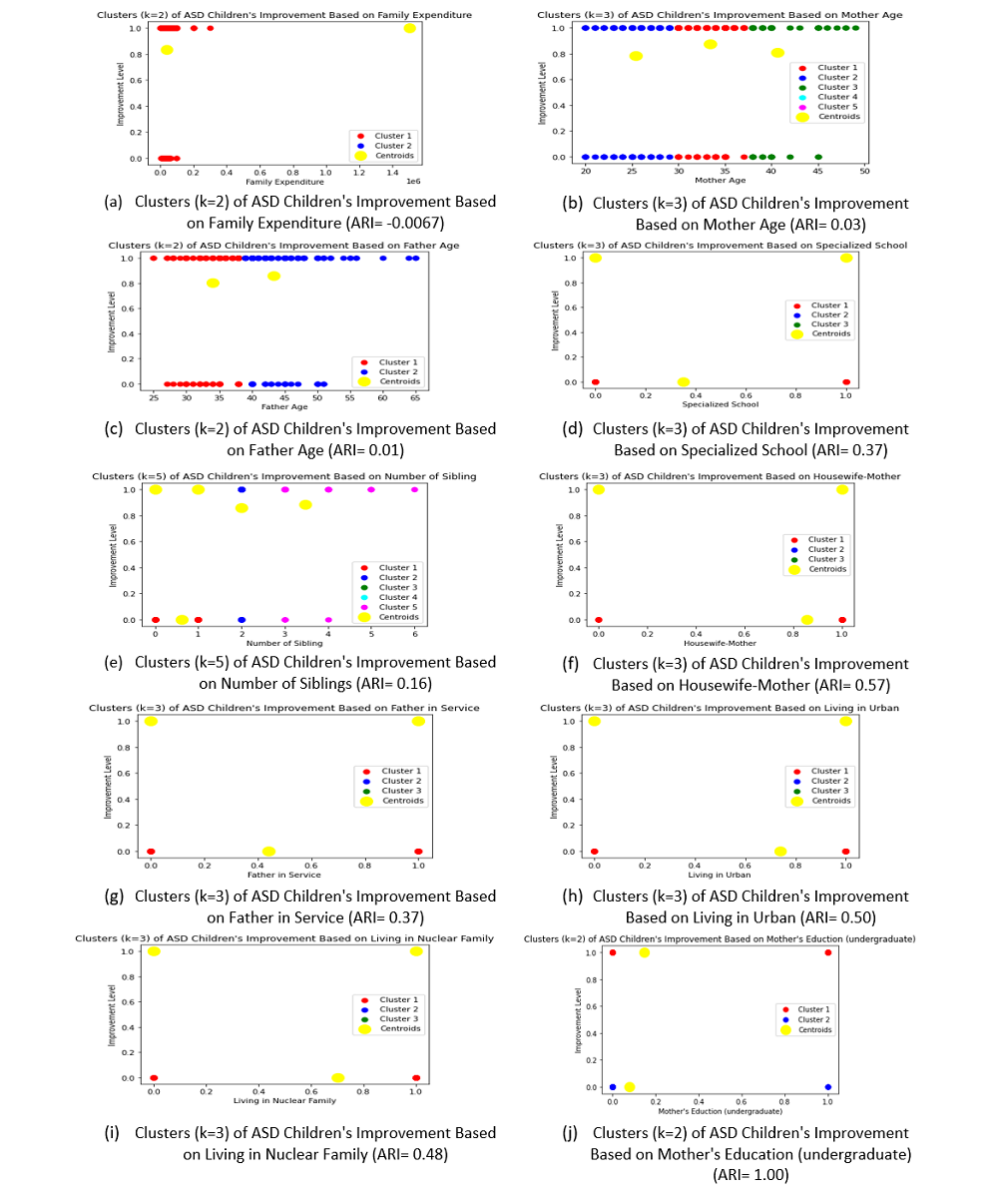
Cluster for the Selected Features of “Daily Living Skills” using K-Means Algorithm. ARI: Adjusted Random Index; ASD: Autism spectrum disorder.

To understand the relation of the 10 selected features (described in the “Feature Selection” section) with the improvement level of “daily living skills” of children with ASD, we implemented K-means clustering [65] to create clusters. As our improvement level is “0” and “1,” we have to describe the children’s improvement clusters by the “cluster centroid.” Figure 2 shows the 10 clusters for the 10 selected features in “daily living skills.” We have selected the cluster number (k) by using the “elbow method” [66]. All elbow graphs are shown in Multimedia Appendix 5. We also validated the cluster number by “Adjusted Random Index” [67].

From the cluster in Figure 2A, we can see that the improvement of children with ASD from high-income families is better than those from low-income families. Age of parents is an important factor in the development of children with ASD, as middle-aged mothers (from Figure 2B) and old-aged fathers (Figure 2C) can take better care of their children’s development. We also obtained similar types of clusters from Figure 2F and 2G, where occupation of parents plays a vital role in the development of their children with ASD. The number of siblings, living in the urban area, and family size (nuclear) are also important factors in our data set. From the clusters in Figure 2E, 2H, and 2I, we can see that small families with less siblings in the urban area can help improve the children in their “daily living skills.” Education levels of children with ASD, especially in specialized school, and their parent’s education, especially mother’s higher education, can also be helpful for their “daily living skills” development (Figure 2D and 2J).

From the explanation of the clusters in Figure 2, we can find the association between our selected feature and the development of children with ASD. Further, using these data, we can validate our main findings, which is described in detail in the “Principal Results” section.

### Building the Model by Machine Learning

We have used 4 supervised machine learning algorithms (decision tree [68], logistic regression [36,69,70], KNN [71,72], and ANN [73-75]) to build the prediction model and compared the results to find out the best machine learning algorithm that can be used for the prediction from this kind of problem and data sets. We used 4 data sets (described in the “Select the Data Set” section) for each algorithm. We used 80% of data for training purposes and 20% for testing purposes from every data set for all the algorithms. We validated our models by k-fold cross-validation (where k=5) [76,77] and took the score’s average as the model’s accuracy. We describe the models based on different machine learning algorithms in the following sections.

### Supervised Machine Learning

#### Decision Tree

For implementation of the decision tree classification algorithm, we used the tree.DecisionTreeClassifier [78] from the sklearn library [79] of Python [80] to build models for 4 distinguished data sets. The highest accuracy (87.85%; average of fivefold cross-validation score) was obtained for the *Brushes teeth* data set among the 4 models. These models were implemented in Python’s Jupyter Notebook [81].

#### Logistic Regression

For implementation of the classification model, we used the LogisticRegression class [82] from the sklearn library [79] of Python [80] to build 4 predictive models from the “daily living skills” milestone parameter. We calculated the accuracy of the model based on the average fivefold cross-validation score, with accuracies for *Brushes teeth*, *Asks to use toilet*, *Urinates in toilet or potty*, and *Buttons large buttons in front, in correct buttonholes* being 95.00%, 77.35%, 84.98%, and 71.55%, respectively.

#### K-Nearest Neighbor

**Figure 3:**
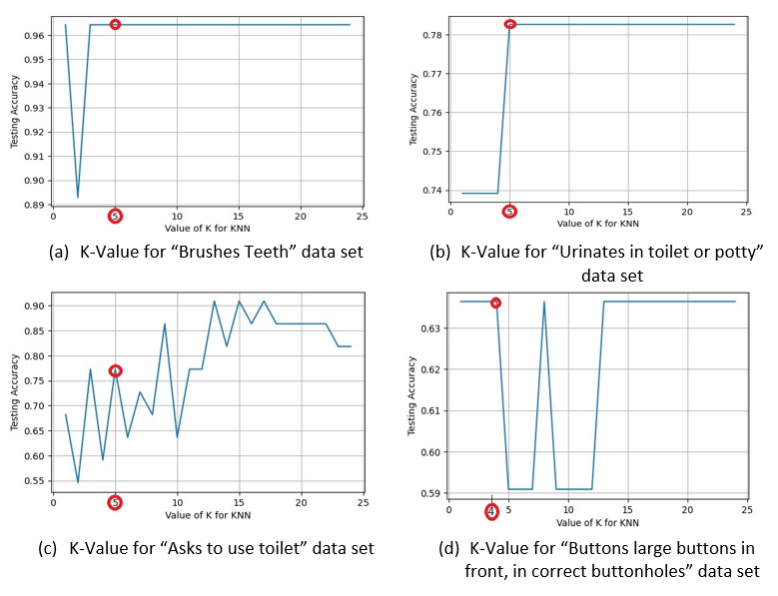
Graphical representation for calculating the best K- value against the test accuracy for the datasets. KNN: K-Nearest Neighbor.

We implemented this model in Python Jupyter Notebook using the KNeighborsClassifier [83] from the sklearn library [79] of Python [80]. In this algorithm, the K-value selection is the key to measure the model’s performance. For this reason, to build the relationship between the K-value and testing accuracy, we created a plot for a range of K-values against the accuracy for every data set (Figure 3). From the graphical representation, we can easily pick the right K-value for a standard accuracy data set. For example, from Figure 3A, we have chosen K=5 for the *Brushes teeth* data set and applied it in the KNeighborsClassifier [83], which created 95.00% (average fivefold cross-validation score) of the model. For other data sets, similarly, we used the K-value from the graphical representation of Figure 3 and obtained satisfactory accuracy (details of outcomes are described in the “Results” section).

#### Artificial Neural Network

**Figure 4:**
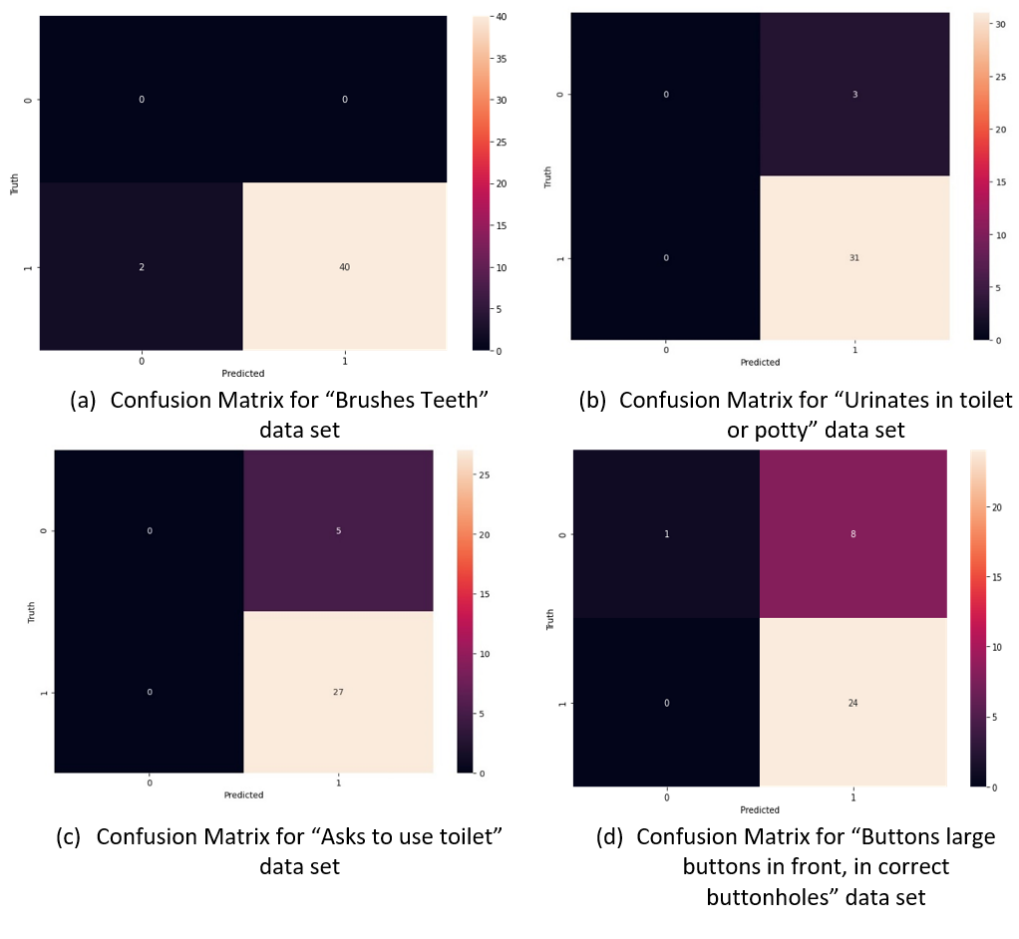
Confusion Matrix for all the Datasets.

We have used the keras.Sequential [84] model from the TensorFlow [85] library to build the models. Figure 4 shows the confusion matrix for the 4 data sets using ANN. Table 4 shows the ANN model’s overall classification report for all data sets.

**Table 4:** The artificial neural network model’s overall classification report for all data sets.

Data set and classification report		Precision	Recall	F1 score	Support
**Brushes teeth**					
	0	0.00	0.00	0.00	0
	1	1.00	0.95	0.98	42
	Accuracy	N/A^a^	N/A	0.95	42
	Macro average	0.50	0.48	0.49	42
	Weighted average	1.00	0.95	0.98	42
**Urinates in toilet or potty**					
	0	0.00	0.00	0.00	3
	1	0.91	1.00	0.95	31
	Accuracy	N/A	N/A	0.91	34
	Macro average	0.46	0.50	0.48	34
	Weighted average	0.83	0.91	0.87	34
**Asks to use toilet**					
	0	0.00	0.00	0.00	5
	1	0.84	1.00	0.92	27
	Accuracy	N/A	N/A	0.84	32
	Macro average	0.42	0.50	0.46	32
	Weighted average	0.71	0.84	0.77	32
**Buttons large buttons in front, in correct buttonholes**					
	0	1.00	0.11	0.20	9
	1	0.75	1.00	0.86	24
	Accuracy	N/A	N/A	0.76	33
	Macro average	0.88	0.56	0.53	33
	Weighted average	0.82	0.76	0.68	33

^a^N/A: not applicable.

## Results

In this study, we have implemented 4 supervised machine languages to build predictive models for the “daily living skill” milestone parameter of children with ASD based on their demography. A summary of the results for different machine learning algorithms for predicting this milestone parameter is presented in Table 5.

We validated the model’s result by a fivefold validation score. From Table 5, we can conclude that, based on the demography, *Daily living skills* and *Brushes teeth* data sets had the highest accuracy in all machine learning–based models. The “ANN” performed well among the machine learning algorithms studied. In conclusion, if we need to develop an automated system to predict the “daily living skill” milestone parameter development based on the demography, then from this study’s outcome, we can recommend developing a system based on machine learning algorithm, especially ANN.

We validated the performance of our classifiers by receiver operating characteristic–area under the curve (ROC–AUC) [86] scores (Table 6), with score “1” considered the outstanding classifier. Rice and Harris [87] suggested that, in applied psychology and prediction model of future behavior, the ROC–AUC values of 0.70 or higher would be considered to have strong effects. The average ROC–AUC scores (from 4 parameters) of the decision tree, logistic regression, KNN, and ANN were 0.84, 0.86, 0.76, and 0.83, respectively (Table 6). The ROC curves of these classifiers are presented in Multimedia Appendices 6-9.

**Table 5:** Summary of the accuracy of all prediction models based on demography for "daily living skills."

Parameter types	Decision tree (fivefold cross-validation score)	Logistic regression (fivefold cross-validation score)	K-nearest neighbor fivefold cross-validation score)	Artificial neural network
Brushes teeth	87.85%	95.00%	95.00% (K=5)	95.00%
Asks to use toilet	71.64%	77.35%	84.00% (K=13)	84.00%
Urinates in toilet or potty	72.52%	84.98%	85.02% (K=5)	91.00%
Buttons large buttons in front, in correct buttonholes	73.46%	71.55%	66.88% (K=5)	76.00%

**Table 6:** Summary of receiver operating characteristic–area under the curve for all prediction models based on demography for "daily living skills."

Parameter types	Decision tree	Logistic regression	K-nearest neighbor	Artificial neural network
Brushes teeth	0.68	0.91	0.65	0.80
Asks to use toilet	0.95	0.77	0.77	0.76
Urinates in toilet or potty	0.78	0.89	0.86	0.91
Buttons large buttons in front, in correct buttonholes	0.94	0.86	0.75	0.84

## Discussion

### Principal Findings

This study reports on some major evidence-based findings regarding patients with ASD and their development in the milestone categories based on demography.

### Finding 1

Among the 4 major milestone categories, “daily living skills” had the highest improvement level. Thus, it can be concluded that the caregiver and care practitioner give more importance to developing the daily living skills of children with ASD so that they can live independently without requiring any help from others.

### Finding 2

**Figure 5:**
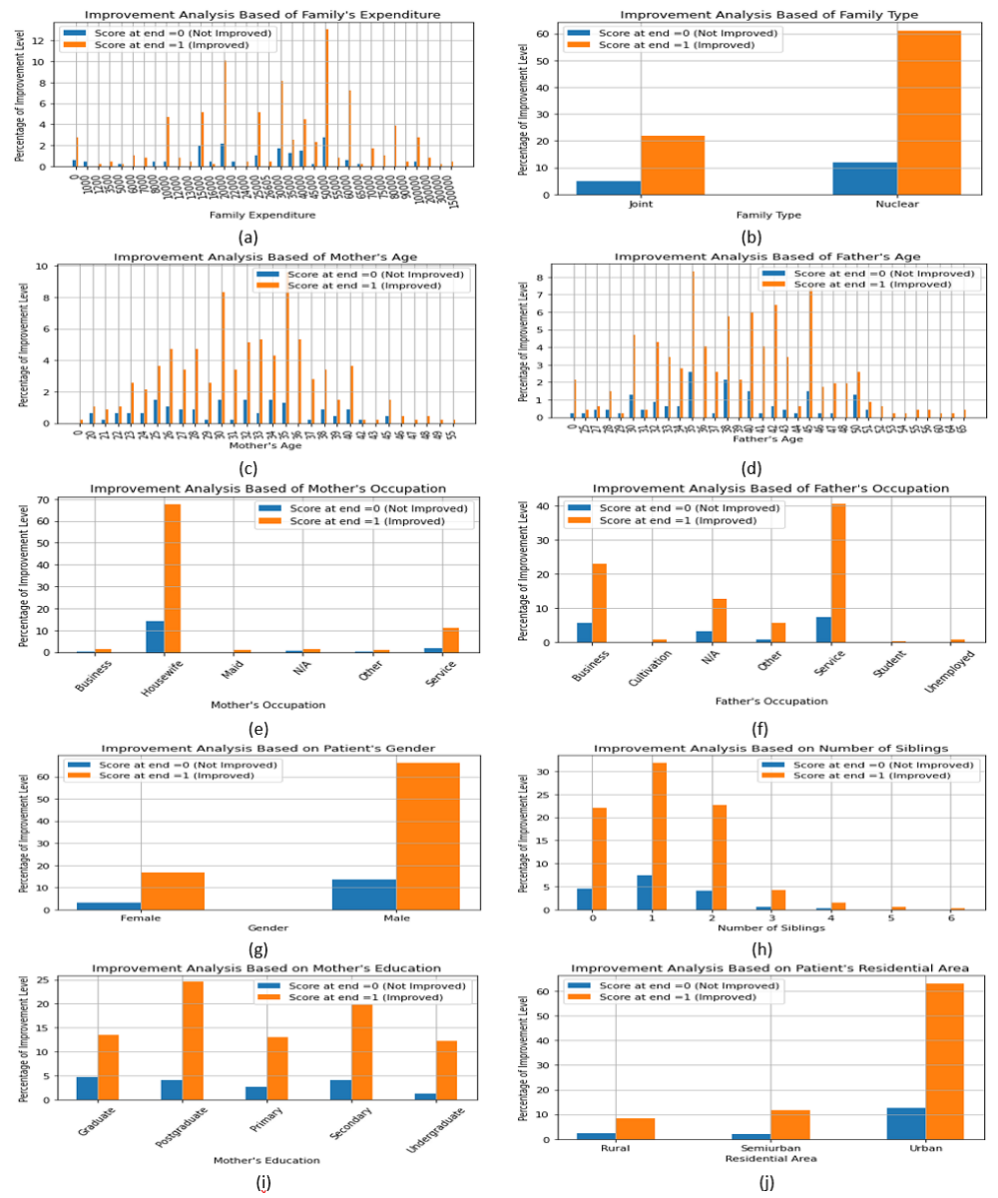
The Summary of the Demography’s importance behind the ASD Children’s Milestone Parameter Development.

The demography of children with ASD impacts the development of their milestone parameters. In Figure 5, we have summarized the demography that impacts the development of their “daily living skills” parameter. Here, “score_at_end”=1 is the final improvement point of the children with ASD. We see that family income or expenditure (Figure 5A) in the middle range helps children with ASD to develop. Besides, a nuclear family (Figure 5B) with a small number of siblings (Figure 5H) in the urban area (Figure 5J) shows the higher improvement rate of children with ASD. The age of parents is also an important factor in the development of children with ASD; generally, middle-aged (aged 25-45) parents can take better care of their children during the course of their development (Figure 5C and 5D). Occupation and education of parents are other good factors to consider; our results show that a mother who works in the house (Figure 5E) but has good education (Figure 5I) and an employed father (Figure 5F) can help achieve significant development in their child. Lastly, gender of patients remains another significant demography in our study, with male children’s development being far better than that of female children (Figure 5G).

### Finding 3

We implemented 4 supervised machine learning algorithms to predict the “daily living skills” improvement level of children with ASD based on their demography. Among the 4 algorithms, the ANN performs better than others, and it has, on average, an average accuracy of over 80% from the same data set we have used in other algorithms. Thus, we can conclude and recommend the ANN to develop a demography-based prediction tool in the intervention or treatment process of children with ASD.

### Limitations

Although we achieved some satisfactory results and reported important findings in this study, our data set lacks in some aspects. The first limitation of the data set is its scattered property, which makes it challenging to find patterns for analysis, but still we achieved good accuracy from this data set. Increasing the number of data can help resolve this problem. Although some studies had been done in this area, the real data set remains very rare. Therefore, we could not compare our study results and findings with other studies and data sets.

### Comparison With Prior Work

Most mental health work is related to identification or recognition and symptom analysis of ASD [88]. In this study, we have implemented machine learning models to predict the improvement level of children with ASD based on their demography. A few studies have been performed in this area, and these are described in the following section.

Scheer et al [38] built a clinical model to predict proximal junctional kyphosis and proximal junctional failure. They used the baseline demographic, radiographic, and surgical factors for 510 patients to build the prediction model. The model’s overall accuracy was 86.3%, which has a great significance in caregiving decision making, risk analysis, and risk prediction before surgery. To build this model, they used the decision tree machine learning algorithm with 5 different bootstrapped models. This model would have been more sophisticated had they used more than 1 machine learning model for the prediction.

Another machine learning–based work has been performed by Tariq et al [2] to detect developmental delay in patients with autism, wherein they used home videos of Bangladeshi children to train and validate the model. Their study’s main objective was to determine the “risk scores” for autism. Using a 2-classification layer neural network, they achieved 85% accuracy for predicting developmental delay. This work has been very effective not only for predicting developmental delay but also for early detection of autism remotely. The authors trained the model with the US data set, but they achieved only low accuracy when applying the Bangladeshi data set. Thus, the model had no cultural divergence.

To evaluate the ADDM status of children, Maenner et al [39] have developed a machine learning–based model using the words and phrases in children’s developmental evaluation. This model has been built with the random forest classifier by deploying the *2008 Georgia* data set containing data on 1162 children. With 86.5% accuracy, the machine learning–based algorithm significantly differentiated between the children that do and do not meet ASD surveillance criteria. As is the case with Scheer et al [38], this work would have been more in-depth had there been more than 1 machine learning algorithm for building the model.

Nowell et al [15] summarized in their review that patients’ demographic has an influence on their ASD development. The main finding of their study was that “myriad demographic factors influence the diagnosis of ASD.” Their study proves that the patient’s demography, including race, socioeconomic status, ethnicity, and parental education, is the most important factor in ASD diagnosis. However, most of the studies reviewed were based on children in the United States.

A sufficient number of studies have been performed to detect ASD by both supervised [2,89-97] and unsupervised machine [98,99] learning methods. In our study, supervised machine learning has mainly been used for the detection of ASD through behavioral or neuroimaging data, whereas unsupervised machine learning was deployed for predicting ASD assessment. In supervised machine learning, logistic regression, KNN, neural network, convolution neural network, naive Bayes, support vector machine, and rule-based machine learning models have been used to detect ASD. Raj and Masood [89] deployed some supervised machine learning models with 3 nonclinical ASD data sets to predict and analyze the problem of ASD. Feature selection–based machine learning has been used to detect ASD with accuracy greater than 90% [90]. Tariq et al [2,91] used home videos of Bangladeshi children with ASD in supervised machine learning to detect their speech and language problems. Küpper et al [92] deployed the clinical behavioral feature in support vector machine to detect the ASD problems in adolescents. Besides these studies, rule-based [93] classification approaches such as decision trees, random forest, and linear discriminant analysis [94-97] have been used to detect ASD. By contrast, unsupervised machine learning has been used for predicting ASD assessment or analysis of ASD problem in children [98,99].

### Comparison With Our Study

Most of the work on children with ASD concerned generalized development, but in this study, we developed prediction models for specific milestone parameters concerning development in children with ASD. Unlike other previous studies, we have validated the prediction result for a specific milestone parameter with more than 1 machine learning algorithm. Our study used the same cultural demographic data set (from Bangladesh) for both training and predicting the models, which helps to get an accurate result from the models.

### Conclusions

This study implies 3 significant factors in the area of mental health development of children with ASD in low- and middle-income countries such as Bangladesh. First, we evaluated the improvement in milestone parameters in children with ASD from the mCARE project. The “daily living skills” and “motor skills” had significant improvement after deploying mCARE tools. We have developed 4 supervised machine learning models based on the demographic information of children with ASD to predict their “daily living skills” development. By comparing the accuracy of the algorithms, we can conclude that the ANN with 1 hidden layer can provide the appropriate prediction for the improvement in “daily living skills” of children with ASD. At the end of the study, from the supervised and unsupervised algorithms, we found some important demographic characteristics that can impact the improvement level in children with ASD. In conclusion, successful and accurate prediction tools deploying this study’s findings will make a renovation in the area of mental health, especially in the development of children with ASD.

## Appendix

Multimedia Appendix 1 Data set features: 18 features about the participant’s demographic for this study.

Multimedia Appendix 2 Best 10 features from “daily living skill” by univariate selection method.

Multimedia Appendix 3 Top 10 Features from “Daily Living Skills” by Feature Importance method.

Multimedia Appendix 4 Correlation Matrix with Heatmap for “Daily Living Skill” Dataset.

Multimedia Appendix 5 Cluster Analysis by “Elbow Method” for the Selected Features of “Daily Living Skills”.

Multimedia Appendix 6 ROC curve for four parameters of “Daily Living Skills” by “Decision Tree” model.

Multimedia Appendix 7 ROC curve for four parameters of “Daily Living Skills” by “Logistic Regression” model.

Multimedia Appendix 8 ROC curve for four parameters of “Daily Living Skills” by “K-Nearest Neighbor” model.

Multimedia Appendix 9 ROC curve for four parameters of “Daily Living Skills” by “Artificial Neural Network” model.

## Abbreviations

ADDM: Autism and Developmental Disabilities Monitoring
ANN: artificial neural network
ASD: autism spectrum disorder
AUC: area under the curve
AWF: Autism Welfare Foundation
IPNA: Institute of Pediatric Neuro-disorder & Autism
KNN: K-nearest neighbor
NIH: National Institutes of Health
NIMH: National Institute of Mental Health
ROC: receiver operating characteristic
