# Supplementary material for: Informing Developmental Milestone Achievement for Children With Autism: Machine Learning Approach
Source: JMIR Med Inform. 2021 Jun 8;9(6):e29242. doi: 10.2196/29242 (PMC8262602; doi:10.2196/29242)
Supplement: Multimedia Appendix 1 [file medinform_v9i6e29242_app1.docx]

| Serial | Participant’s Demographic | Serial | Participant’s Demographic |
| --- | --- | --- | --- |
| 1 | Mother Age | 10 | ASD Children Education |
| 2 | Mother Died Age | 11 | Residential Area |
| 3 | Mother Education | 12 | Live in own House |
| 4 | Mother Occupation | 13 | Family Type |
| 5 | Father Age | 14 | Number of Siblings |
| 6 | Father Died Age | 15 | Family Expenditure |
| 7 | Father Education | 16 | Mental Illness History |
| 8 | Father Occupation | 17 | Born Complication |
| 9 | Gender | 18 | History of Convulsion |
