# Supplementary material for: Informing Developmental Milestone Achievement for Children With Autism: Machine Learning Approach
Source: JMIR Med Inform. 2021 Jun 8;9(6):e29242. doi: 10.2196/29242 (PMC8262602; doi:10.2196/29242)
Supplement: Multimedia Appendix 2 [file medinform_v9i6e29242_app2.docx]

| Specs | Score |
| --- | --- |
| 5 family_expenditure | 343642.6 |
| 3 father_died_age | 68.2 |
| 1 mother_died_age | 9.5 |
| 29 never_s | 6.7 |
| 8 m_graduate | 5.3 |
| 0 mother_age | 3.4 |
| 30 specialized_s | 2.8 |
| 2 father_age | 2.4 |
| 10 m_undergraduate | 2.2 |
| 12 f_secondary | 1.4 |
