# Supplementary figures and images for: Informing Developmental Milestone Achievement for Children With Autism: Machine Learning Approach
Source: JMIR Med Inform. 2021 Jun 8;9(6):e29242. doi: 10.2196/29242 (PMC8262602; doi:10.2196/29242)

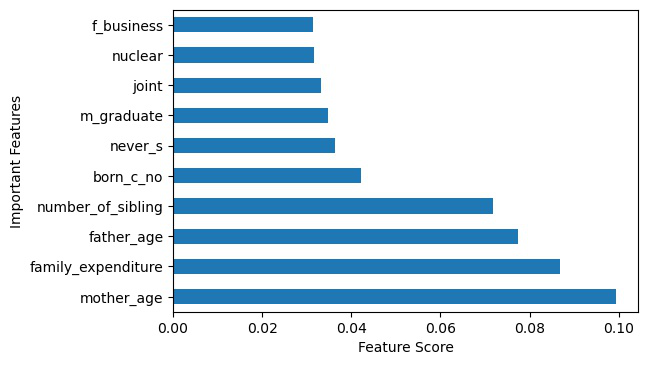

Supplement: Multimedia Appendix 3 [file medinform_v9i6e29242_app3.png]

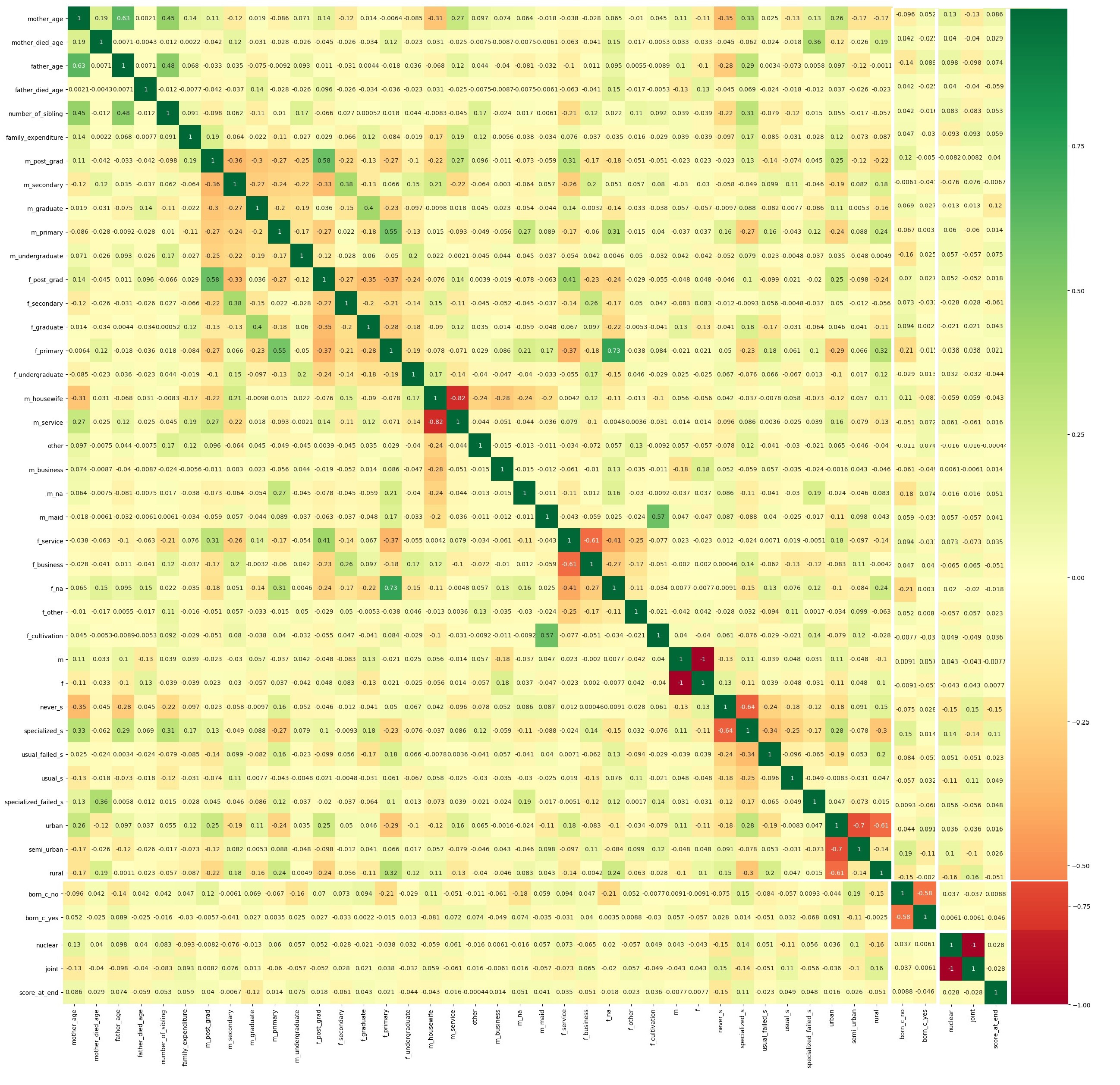

Supplement: Multimedia Appendix 4 [file medinform_v9i6e29242_app4.png]

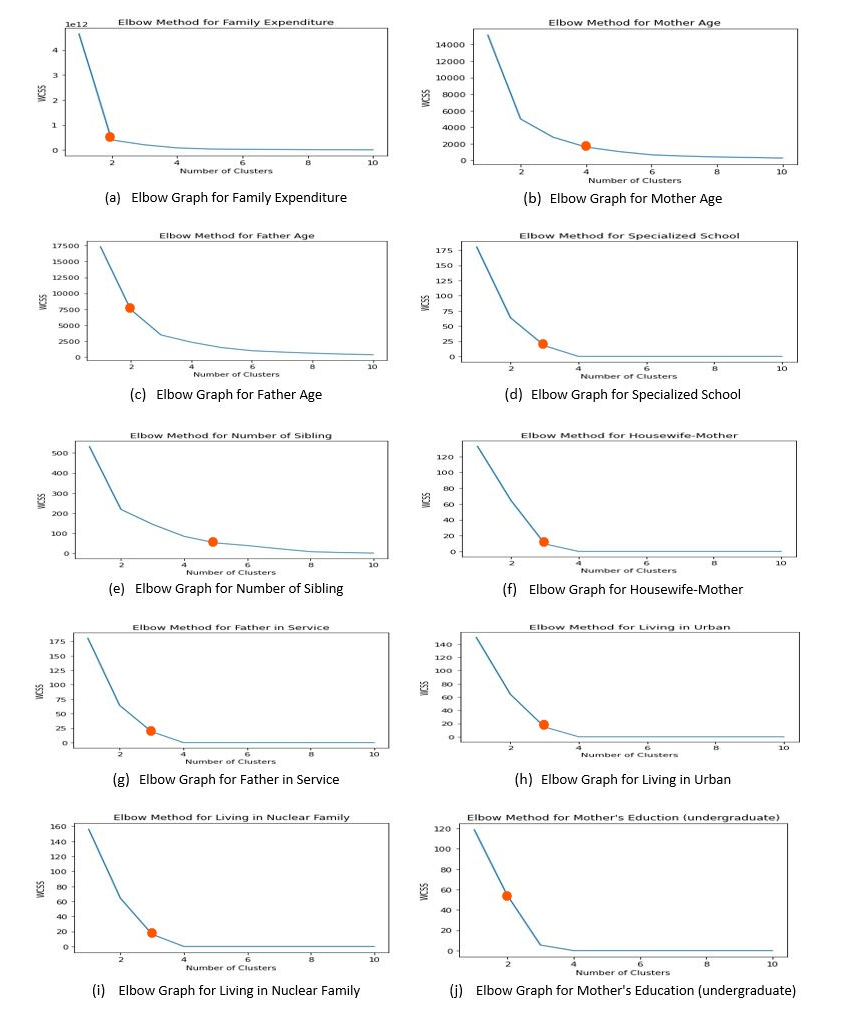

Supplement: Multimedia Appendix 5 [file medinform_v9i6e29242_app5.png]

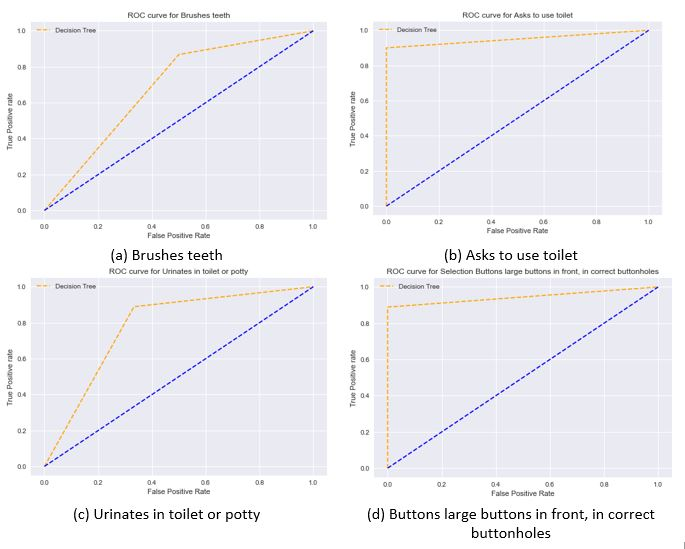

Supplement: Multimedia Appendix 6 [file medinform_v9i6e29242_app6.png]

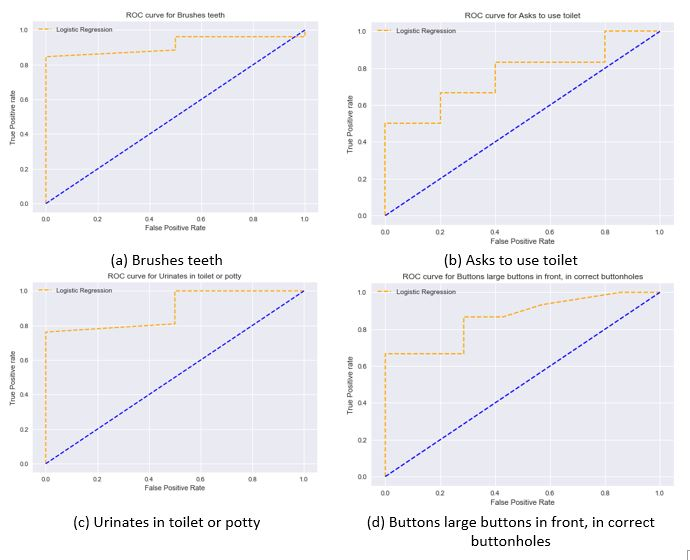

Supplement: Multimedia Appendix 7 [file medinform_v9i6e29242_app7.png]

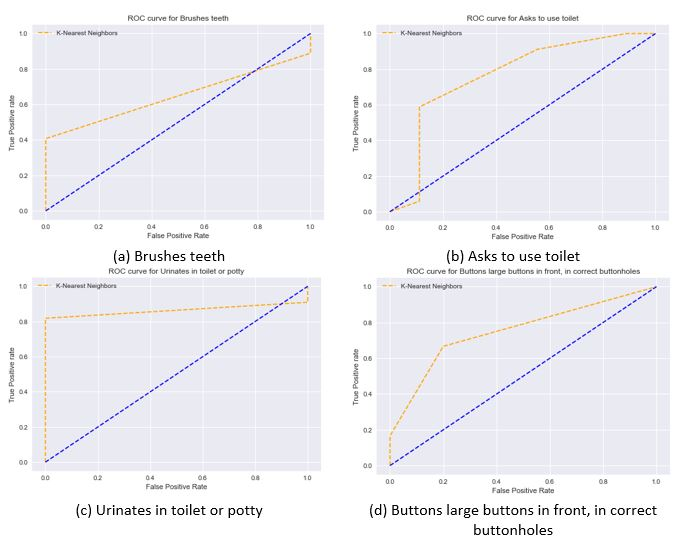

Supplement: Multimedia Appendix 8 [file medinform_v9i6e29242_app8.png]

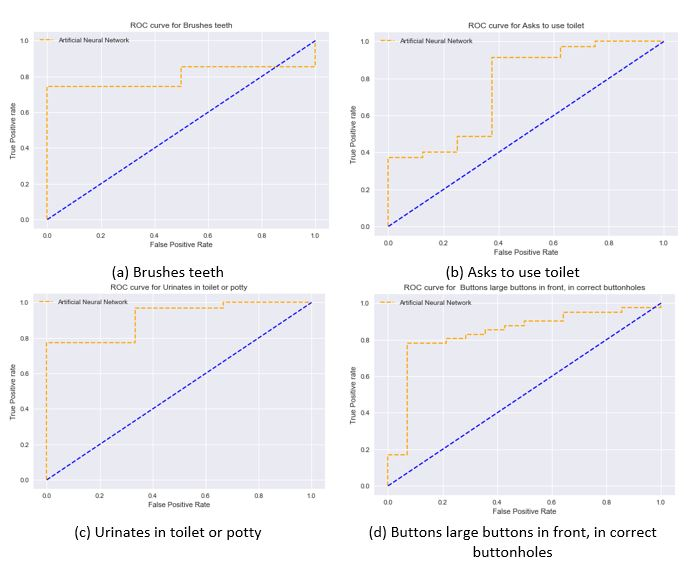

Supplement: Multimedia Appendix 9 [file medinform_v9i6e29242_app9.png]
